# Supplementary material for: Automatic classification between COVID-19 pneumonia, non-COVID-19 pneumonia, and the healthy on chest X-ray image: combination of data augmentation methods
Source: Sci Rep. 2020 Oct 16;10:17532. doi: 10.1038/s41598-020-74539-2 (PMC7567783; doi:10.1038/s41598-020-74539-2)
Supplement: Supplementary file 1 — Supplementary Information. [file 41598_2020_74539_MOESM1_ESM.docx]

**Supplementary information**

**Full Title**

Automatic classification between COVID-19 pneumonia, non-COVID-19 pneumonia, and the healthy on chest X-ray image: combination of data augmentation methods

**Authors and Affiliations**

Mizuho Nishio, MD, PhD^1^, Shunjiro Noguchi, MD^2^, Hidetoshi Matsuo, MD^1^, Takamichi Murakami, MD, PhD^1^

1 Department of Radiology, Kobe University Graduate School of Medicine, 7-5-2 Kusunoki-cho, Chuo-ku, Kobe 650-0017 JAPAN

2 Department of Diagnostic Imaging and Nuclear Medicine, Kyoto University Graduate School of Medicine, 54 Shogoin Kawaharacho, Sakyo-ku, Kyoto, 606-8507, Japan

Doc S1

Summary of optimal VGG16-based model. Note: This summary was obtained by model.summary() of Keras. Input, Maxpooling2D, GlobalAveragePooling2D, and Dropout layers have no trainable parameters. Conv2D and Dense represent convolutional layer and fully-connected layer, respectively.

Layer (type) Output Shape Param #

========================================================================================================================

input_5 (InputLayer) [(None, 220, 220, 3)] 0

________________________________________________________________________________________________________________________

block1_conv1 (Conv2D) (None, 220, 220, 64) 1792

________________________________________________________________________________________________________________________

block1_conv2 (Conv2D) (None, 220, 220, 64) 36928

________________________________________________________________________________________________________________________

block1_pool (MaxPooling2D) (None, 110, 110, 64) 0

________________________________________________________________________________________________________________________

block2_conv1 (Conv2D) (None, 110, 110, 128) 73856

________________________________________________________________________________________________________________________

block2_conv2 (Conv2D) (None, 110, 110, 128) 147584

________________________________________________________________________________________________________________________

block2_pool (MaxPooling2D) (None, 55, 55, 128) 0

________________________________________________________________________________________________________________________

block3_conv1 (Conv2D) (None, 55, 55, 256) 295168

________________________________________________________________________________________________________________________

block3_conv2 (Conv2D) (None, 55, 55, 256) 590080

________________________________________________________________________________________________________________________

block3_conv3 (Conv2D) (None, 55, 55, 256) 590080

________________________________________________________________________________________________________________________

block3_pool (MaxPooling2D) (None, 27, 27, 256) 0

________________________________________________________________________________________________________________________

block4_conv1 (Conv2D) (None, 27, 27, 512) 1180160

________________________________________________________________________________________________________________________

block4_conv2 (Conv2D) (None, 27, 27, 512) 2359808

________________________________________________________________________________________________________________________

block4_conv3 (Conv2D) (None, 27, 27, 512) 2359808

________________________________________________________________________________________________________________________

block4_pool (MaxPooling2D) (None, 13, 13, 512) 0

________________________________________________________________________________________________________________________

block5_conv1 (Conv2D) (None, 13, 13, 512) 2359808

________________________________________________________________________________________________________________________

block5_conv2 (Conv2D) (None, 13, 13, 512) 2359808

________________________________________________________________________________________________________________________

block5_conv3 (Conv2D) (None, 13, 13, 512) 2359808

________________________________________________________________________________________________________________________

block5_pool (MaxPooling2D) (None, 6, 6, 512) 0

________________________________________________________________________________________________________________________

global_average_pooling2d_4 (GlobalAveragePooling2D) (None, 512) 0

________________________________________________________________________________________________________________________

dense_5 (Dense) (None, 416) 213408

________________________________________________________________________________________________________________________

dropout_1 (Dropout) (None, 416) 0

________________________________________________________________________________________________________________________

dense_6 (Dense) (None, 3) 1251

========================================================================================================================

Table S1

Effect of RICAP as data augmentation method

| Model | Loss of test set |
| --- | --- |
| proposed method (conventional data augmentation method and mixup with layer freezing) | 0.4682 |
| conventional data augmentation method and RICAP with layer freezing | 0.4724 |
| conventional data augmentation method, mixup, and RICAP with layer freezing | 0.4761 |

Note: Value of each cell was mean of 5 trials.
